# Supplementary material for: Large-scale biometry with interpretable neural network regression on UK Biobank body MRI
Source: Sci Rep. 2020 Oct 20;10:17752. doi: 10.1038/s41598-020-74633-5 (PMC7576214; doi:10.1038/s41598-020-74633-5)
Supplement: Supplementary file 1 — Supplementary Information. [file 41598_2020_74633_MOESM1_ESM.pdf]

Supplementary Material

Title: Large-scale biometry with interpretable neural network regression on UK Biobank body MRI

Authors: Taro Langner, Robin Strand, Håkan Ahlström, Joel Kullberg

Evaluation details for all remaining targets are given in Supplementary Tables 1, 2, 3, and 4, whereas Supplementary Table 5 lists Pearson's correlation coefficients for the similarity between measurements of symmetrical body parts to each other and to their predictions by the network.

Supplementary Table 1. Inference of main fields

field	name	N	unit	[min , max]	mean ± SD	MAE	LoA	R^2	R^2_{lr}
	**DXA:**								
23279	TotalFatFreeMass	4544	kg	[7.5 , 84.3]	49.9 ± 10.2	0.79	(-2.05 to 1.98)	0.990	0.908
23278	TotalFatMass	4544	kg	[2.7 , 76.1]	26.1 ± 9.0	0.56	(-1.36 to 1.59)	0.993	0.894
23280	TotalLeanMass	4544	kg	[6.8 , 80.3]	47.3 ± 9.7	0.82	(-2.07 to 2.11)	0.988	0.904
23281	TotalTissueFat	4544	%	[11.8 , 58.4]	35.2 ± 8.0	0.67	(-1.67 to 1.68)	0.988	0.740
23282	TotalTissueMass	4544	kg	[9.5 , 154.0]	73.3 ± 14.5	0.79	(-2.07 to 2.17)	0.994	0.993
23289	VatVolume	4498	L	[0.0 , 6.6]	1.3 ± 1.0	0.12	(-0.30 to 0.33)	0.972	0.718
	**MRI:**								
22403	AnteriorThighMuscleR	5662	L	[0.6 , 3.7]	1.7 ± 0.5	0.06	(-0.15 to 0.15)	0.975	0.796
22404	PosteriorThighMuscleR	5662	L	[1.4 , 6.2]	3.4 ± 0.8	0.08	(-0.21 to 0.21)	0.983	0.829
22405	AnteriorThighMuscleL	5607	L	[0.7 , 3.6]	1.7 ± 0.5	0.06	(-0.14 to 0.15)	0.975	0.802
22406	PosteriorThighMuscleL	5607	L	[1.3 , 6.3]	3.4 ± 0.8	0.08	(-0.22 to 0.21)	0.981	0.824
22407	VatVolume	5763	L	[0.1 , 14.4]	3.8 ± 2.2	0.14	(-0.37 to 0.39)	0.993	0.703
22408	AsatVolume	5763	L	[1.5 , 23.5]	7.1 ± 3.1	0.22	(-0.62 to 0.61)	0.990	0.822
22409	TotalThighMuscle	5559	L	[4.3 , 19.0]	10.2 ± 2.5	0.18	(-0.43 to 0.46)	0.992	0.846
22410	TotalTrunkFat	5763	L	[1.9 , 31.6]	10.9 ± 4.5	0.24	(-0.68 to 0.63)	0.994	0.843
22415	TotalAdiposeTissue	8276	L	[5.5 , 65.9]	21.1 ± 7.0	0.37	(-0.99 to 1.07)	0.994	0.879
22416	TotalLeanTissue	8276	L	[12.3 , 43.3]	24.2 ± 4.8	0.64	(-1.92 to 1.69)	0.963	0.846
	**Other:**								
48	waist	30441	cm	[55.0 , 184.0]	88.6 ± 12.6	3.35	(-8.70 to 8.10)	0.883	0.815
49	hip	30443	cm	[72.0 , 157.0]	101.3 ± 8.6	2.60	(-6.60 to 6.61)	0.847	0.759
21001	BMI	30124	kg/m^2	[14.2 , 62.0]	26.6 ± 4.3	0.41	(-1.13 to 0.99)	0.984	0.969
22402	liverFat	4419	%	[0.0 , 46.0]	4.0 ± 4.7	1.35	(-4.04 to 4.22)	0.799	0.208
47	gripStrengthRight	30053	kg	[-0.0 , 72.0]	31.3 ± 10.5	5.08	(-12.92 to 12.87)	0.607	0.583
102	pulseRate	25123	bpm	[33.0 , 157.0]	69.4 ± 12.1	8.10	(-20.13 to 20.76)	0.262	0.058

*SD: Standard deviation, MAE: Mean absolute error, LoA: Limits of agreement.
R^2_{lr}: Fit of multiple linear regression on age, sex, height and weight.

Supplementary Table 2. Inference of DXA trunk fields

field	name	N	unit	[min, max]	mean ± SD	MAE	LoA	R^2	R^2_{lr}
	**Android:**								
23244	BoneMass	4544	g	[16.0, 118.0]	49.0 ± 13.0	5.62	(-14.28 to 14.80)	0.676	0.411
23245	FatMass	4544	kg	[0.2, 9.4]	2.5 ± 1.2	0.11	(-0.30 to 0.32)	0.982	0.835
23246	LeanMass	4544	kg	[0.3, 6.8]	3.5 ± 0.8	0.14	(-0.34 to 0.35)	0.945	0.833
23247	TissueFat	4544	%	[8.1, 65.7]	39.7 ± 10.5	1.29	(-3.23 to 3.30)	0.975	0.603
23248	TotalMass	4544	kg	[0.5, 16.4]	6.0 ± 1.7	0.20	(-0.50 to 0.52)	0.975	0.925
	**Gynoid:**								
23261	BoneMass	4544	g	[53.0, 516.0]	274.1 ± 67.1	15.28	(-37.44 to 40.16)	0.912	0.731
23262	FatMass	4544	kg	[0.2, 13.5]	4.2 ± 1.5	0.15	(-0.35 to 0.44)	0.980	0.811
23263	LeanMass	4544	kg	[0.4, 14.1]	7.3 ± 1.6	0.18	(-0.49 to 0.45)	0.977	0.876
23264	TissueFat	4544	%	[11.8, 61.5]	36.1 ± 9.1	0.92	(-2.29 to 2.33)	0.983	0.770
23265	TotalMass	4544	kg	[0.7, 27.5]	11.8 ± 2.2	0.22	(-0.58 to 0.65)	0.980	0.915
	**Trunk:**								
23284	FatMass	4544	kg	[1.2, 46.0]	14.7 ± 5.9	0.46	(-1.22 to 1.22)	0.989	0.857
23285	LeanMass	4544	kg	[2.4, 38.6]	22.8 ± 4.4	0.57	(-1.42 to 1.47)	0.972	0.843
23286	TissueFat	4544	%	[10.5, 62.1]	38.2 ± 9.1	0.96	(-2.24 to 2.53)	0.982	0.647
23287	TotalMass	4544	kg	[4.2, 82.6]	38.3 ± 8.6	0.71	(-1.75 to 1.94)	0.988	0.957

*SD: Standard deviation, MAE: Mean absolute error, LoA: Limits of agreement.
R^2_{lr}: Fit of multiple linear regression on age, sex, height and weight.

Supplementary Table 3. Inference of DXA arm fields

field	name	N	unit	[min, max]	mean ± SD	MAE	LoA	R^2	R^2_{lr}
	**Left arm:**								
23249	FatMass	3834	kg	[0.4, 4.6]	1.3 ± 0.5	0.12	(-0.34 to 0.35)	0.866	0.747
23250	LeanMass	3834	kg	[1.1, 5.5]	2.6 ± 0.8	0.15	(-0.39 to 0.39)	0.936	0.855
23251	TissueFat	3834	%	[11.1, 60.9]	33.9 ± 9.8	1.77	(-4.45 to 4.39)	0.947	0.770
23252	TotalMass	3834	kg	[2.0, 8.3]	4.1 ± 1.0	0.25	(-0.63 to 0.65)	0.885	0.858
	**Right arm:**								
23253	FatMass	3834	kg	[0.4, 4.6]	1.4 ± 0.5	0.12	(-0.35 to 0.33)	0.867	0.744
23254	LeanMass	3834	kg	[1.3, 5.6]	2.8 ± 0.8	0.15	(-0.38 to 0.40)	0.940	0.860
23255	TissueFat	3834	%	[11.0, 60.9]	33.3 ± 9.6	1.68	(-4.16 to 4.33)	0.949	0.770
23256	TotalMass	3834	kg	[2.2, 8.3]	4.3 ± 1.0	0.25	(-0.63 to 0.65)	0.887	0.862
	**Arms, total:**								
23257	FatMass	4544	kg	[0.4, 9.3]	2.7 ± 0.9	0.23	(-0.63 to 0.62)	0.885	0.755
23258	LeanMass	4544	kg	[0.9, 11.0]	5.4 ± 1.6	0.27	(-0.71 to 0.67)	0.951	0.869
23259	TissueFat	4544	%	[11.1, 60.9]	33.6 ± 9.7	1.60	(-3.87 to 4.12)	0.955	0.775
23260	TotalMass	4544	kg	[1.3, 16.6]	8.4 ± 1.9	0.44	(-1.10 to 1.18)	0.907	0.876

*SD: Standard deviation, MAE: Mean absolute error, LoA: Limits of agreement.
R^2_{lr}: Fit of multiple linear regression on age, sex, height and weight.

Supplementary Table 4. Inference of DXA leg fields

field	name	N	unit	[min , max]	mean ± SD	MAE	LoA	R^2	R^2_{lr}
	**Left leg:**								
23266	FatMass	3834	kg	[0.9 , 13.6]	3.9 ± 1.5	0.18	(-0.52 to 0.44)	0.972	0.746
23267	LeanMass	3834	kg	[3.7 , 16.1]	7.9 ± 1.8	0.29	(-0.68 to 0.81)	0.956	0.879
23268	TissueFat	3834	%	[10.7 , 62.0]	32.5 ± 9.5	1.06	(-2.61 to 2.68)	0.980	0.767
23269	TotalMass	3834	kg	[7.0 , 25.6]	12.3 ± 2.4	0.38	(-0.97 to 1.07)	0.952	0.871
	**Right leg:**								
23270	FatMass	3834	kg	[1.0 , 13.5]	3.9 ± 1.5	0.17	(-0.48 to 0.47)	0.974	0.742
23271	LeanMass	3834	kg	[4.0 , 15.4]	8.1 ± 1.9	0.28	(-0.71 to 0.74)	0.960	0.883
23272	TissueFat	3834	%	[10.7 , 61.9]	32.6 ± 9.5	1.02	(-2.57 to 2.56)	0.981	0.763
23273	TotalMass	3834	kg	[6.8 , 25.6]	12.5 ± 2.4	0.36	(-0.92 to 1.01)	0.957	0.875
	**Legs, total:**								
23274	FatMass	4544	kg	[1.0 , 27.1]	7.8 ± 3.0	0.30	(-0.79 to 0.89)	0.979	0.750
23275	LeanMass	4544	kg	[3.4 , 31.5]	16.0 ± 3.7	0.48	(-1.14 to 1.33)	0.970	0.888
23276	TissueFat	4544	%	[10.7 , 62.0]	32.6 ± 9.5	0.92	(-2.19 to 2.42)	0.985	0.768
23277	TotalMass	4544	kg	[4.5 , 52.7]	24.8 ± 4.8	0.63	(-1.81 to 1.53)	0.967	0.880

*SD: Standard deviation, MAE: Mean absolute error, LoA: Limits of agreement.
R^2_{lr}: Fit of multiple linear regression on age, sex, height and weight.

Supplementary Table 5. Symmetrical measurements

$field_a$	$name_a$	$field_b$	$name_b$	N	$r_{(a,b)}$	$r_{(a,net)}$	$r_{(b,net)}$
22405	MriAnteriorThighLeanMuscleLeft	22403	MriAnteriorThighLeanMuscleRight	5559	0.974	**0.988**	**0.987**
22406	MriPosteriorThighLeanMuscleLeft	22404	MriPosteriorThighLeanMuscleRight	5559	0.983	**0.991**	**0.992**
23249	DxaArmFatMassLeft	23253	DxaArmFatMassRight	3834	**0.971**	0.931	0.931
23250	DxaArmLeanMassLeft	23254	DxaArmLeanMassRight	3834	**0.978**	0.968	0.969
23251	DxaArmTissueFatPercentageLeft	23255	DxaArmTissueFatPercentageRight	3834	**0.984**	0.973	0.974
23252	DxaArmTotalMassLeft	23256	DxaArmTotalMassRight	3834	**0.966**	0.941	0.942
23266	DxaLegFatMassLeft	23270	DxaLegFatMassRight	3834	**0.989**	0.986	0.987
23267	DxaLegLeanMassLeft	23271	DxaLegLeanMassRight	3834	**0.984**	0.978	0.980
23268	DxaLegTissueFatPercentageLeft	23272	DxaLegTissueFatPercentageRight	3834	**0.992**	0.990	0.990
23269	DxaLegTotalMassLeft	23273	DxaLegTotalMassRight	3834	**0.982**	0.976	0.979

*Correlations between symmetrical fields and network predictions. The fields (a) and (b) correlate by $r_{(a,b)}$ whereas the network output correlates to field (a) by $r_{(a,net)}$. Only those N subjects were evaluated for whom both measurements were available. Bold font denotes numerically higher values.
